# Rhea, the reaction knowledgebase in 2022

**DOI:** 10.1093/nar/gkab1016

**Published:** 2021-11-10

**Authors:** Parit Bansal, Anne Morgat, Kristian B Axelsen, Venkatesh Muthukrishnan, Elisabeth Coudert, Lucila Aimo, Nevila Hyka-Nouspikel, Elisabeth Gasteiger, Arnaud Kerhornou, Teresa Batista Neto, Monica Pozzato, Marie-Claude Blatter, Alex Ignatchenko, Nicole Redaschi, Alan Bridge

**Affiliations:** Swiss-Prot group, SIB Swiss Institute of Bioinformatics, Centre Medical Universitaire, CH-1211 Geneva 4, Switzerland; Swiss-Prot group, SIB Swiss Institute of Bioinformatics, Centre Medical Universitaire, CH-1211 Geneva 4, Switzerland; Swiss-Prot group, SIB Swiss Institute of Bioinformatics, Centre Medical Universitaire, CH-1211 Geneva 4, Switzerland; Swiss-Prot group, SIB Swiss Institute of Bioinformatics, Centre Medical Universitaire, CH-1211 Geneva 4, Switzerland; Swiss-Prot group, SIB Swiss Institute of Bioinformatics, Centre Medical Universitaire, CH-1211 Geneva 4, Switzerland; Swiss-Prot group, SIB Swiss Institute of Bioinformatics, Centre Medical Universitaire, CH-1211 Geneva 4, Switzerland; Swiss-Prot group, SIB Swiss Institute of Bioinformatics, Centre Medical Universitaire, CH-1211 Geneva 4, Switzerland; Swiss-Prot group, SIB Swiss Institute of Bioinformatics, Centre Medical Universitaire, CH-1211 Geneva 4, Switzerland; Swiss-Prot group, SIB Swiss Institute of Bioinformatics, Centre Medical Universitaire, CH-1211 Geneva 4, Switzerland; Swiss-Prot group, SIB Swiss Institute of Bioinformatics, Centre Medical Universitaire, CH-1211 Geneva 4, Switzerland; Swiss-Prot group, SIB Swiss Institute of Bioinformatics, Centre Medical Universitaire, CH-1211 Geneva 4, Switzerland; Swiss-Prot group, SIB Swiss Institute of Bioinformatics, Centre Medical Universitaire, CH-1211 Geneva 4, Switzerland; EMBL-EBI European Molecular Biology Laboratory, European Bioinformatics Institute (EMBL-EBI), Wellcome Genome Campus, Hinxton, Cambridge CB10 1SD, UK; Swiss-Prot group, SIB Swiss Institute of Bioinformatics, Centre Medical Universitaire, CH-1211 Geneva 4, Switzerland; Swiss-Prot group, SIB Swiss Institute of Bioinformatics, Centre Medical Universitaire, CH-1211 Geneva 4, Switzerland

## Abstract

Rhea (https://www.rhea-db.org) is an expert-curated knowledgebase of biochemical reactions based on the chemical ontology ChEBI (Chemical Entities of Biological Interest) (https://www.ebi.ac.uk/chebi). In this paper, we describe a number of key developments in Rhea since our last report in the database issue of Nucleic Acids Research in 2019. These include improved reaction coverage in Rhea, the adoption of Rhea as the reference vocabulary for enzyme annotation in the UniProt knowledgebase UniProtKB (https://www.uniprot.org), the development of a new Rhea website, and the designation of Rhea as an ELIXIR Core Data Resource. We hope that these and other developments will enhance the utility of Rhea as a reference resource to study and engineer enzymes and the metabolic systems in which they function.

## INTRODUCTION

Rhea (https://www.rhea-db.org) is an expert-curated knowledgebase of biochemical reactions that uses the chemical ontology ChEBI (Chemical Entities of Biological Interest) (https://www.ebi.ac.uk/chebi) (1) to represent reaction participants. Rhea covers enzymatic reactions and transport reactions, including but not limited to those described by the Enzyme Classification of the IUBMB (2,3), as well as reactions that occur spontaneously in biological systems. Rhea is now the reference vocabulary for enzyme annotation in the UniProt Knowledgebase UniProtKB (https://www.uniprot.org) (4–6) and provides reaction data for a host of other resources including the enzyme knowledgebases IntEnz (7) and the Enzyme Portal (8), the metabolomics data repository MetaboLights (9), the lipidomics knowledgebase SwissLipids (10), and the open chemistry database PubChem (11). Rhea reaction data is widely used for the annotation of genomes (12,13) and genome-scale metabolic models (14–19), for integrated analysis of metabolomics data (20), and for computational pathway design (21–23).

In this paper, we highlight a number of key developments in Rhea since our last publication in the database issue of *Nucleic Acids Research* (24). These include improved reaction coverage in Rhea and UniProtKB, the development of a new Rhea website, which provides powerful chemical structure and ontology search facilities to mine Rhea, and the selection of Rhea as an ELIXIR Core Data Resource.

## RESULTS

### Growth of reaction coverage in Rhea

Rhea release 119 of June 2021 describes 13 673 unique reactions involving 11 886 unique reaction participants and evidenced by 15 500 unique literature references (PubMed identifiers), an increase of 2500 curated reactions, 1970 curated reaction participants, and 2889 curated literature references since our last publication on Rhea (which described release 96 of July 2018) (24). Rhea reaction data is curated from the literature by expert biochemists, supported by natural language processing (NLP) tools such as LitSuggest, a web-based system for literature recommendation and curation (25). We have created a LitSuggest model that identifies literature relevant to enzymatic reactions, which we use to provide curators with a weekly digest of newly published literature and to scan all existing literature in MEDLINE, the National Library of Medicine's (NLM) bibliographic database. Tools like LitSuggest will be vital aids to filling our gaps in enzyme knowledge from the scientific literature.

### Rhea annotation in UniProtKB

In 2019, the UniProt Consortium adopted Rhea as the reference vocabulary for enzyme and transporter annotation in the UniProt Knowledgebase UniProtKB (4–6). Rhea and UniProt curators now work closely together, with the Rhea editorial team curating new reactions in response to requests from UniProt curators, who then link those reactions to enzyme and transporter sequences in reviewed protein sequence entries in UniProtKB/Swiss-Prot. The UniProt automatic annotation resource, UniRule, provides Rhea reaction annotations for unreviewed protein sequence records in UniProtKB/TrEMBL (26). Reaction coverage in UniProtKB has grown by around 40% since the first publication describing enzyme annotation in UniProtKB using Rhea (4), up from 6654 Rhea reactions in UniProt release 2019_09 of October 2019 to 9294 reactions in UniProt release 2021_03 of June 2021 (around 68% of 13 673 Rhea reactions). UniProtKB currently provides Rhea annotations for over 222 000 UniProtKB/Swiss-Prot records and 23.2 million UniProtKB/TrEMBL records. These annotations power a range of enhanced chemical structure-based and chemical ontology-based searches over enzymes in UniProtKB via the UniProt website, API and SPARQL endpoint. Interested readers can find more details in the corresponding UniProt publications (4–6).

### Rhea annotation in SwissLipids

In addition to UniProtKB, Rhea also provides reaction data for the SwissLipids knowledgebase (10), which features a library of over 700 000 known and theoretically feasible lipids that is fully mapped to the ChEBI ontology and community standard mass-spectrometry based lipid classifications (27). Rhea curators work closely with SwissLipids curators to capture knowledge of lipid metabolic pathways in ChEBI and Rhea (which SwissLipids curators then use to enumerate libraries of possible lipid structures in SwissLipids), and Rhea currently includes over 4600 reactions involving lipids. SwissLipids and Rhea curators are also working together to 'digitize' the SphinGOMAP resource of sphingolipid pathways (www.sphingomap.org) (28) in ChEBI and Rhea. The SphinGOMAP, originally compiled by Professor Alfred Merrill Jr and collaborators, is an incredible resource of knowledge of sphingolipid pathways in graphical form; mapping this knowledge to ChEBI and Rhea makes it accessible for computation and facilitates the annotation of the corresponding enzymes in UniProtKB.

### Rhea website

The new Rhea website, released in October 2020, provides improved views of reaction data and powerful interactive search tools and programmatic access. We summarize the main features in the following sections, while users can also find further assistance at https://www.rhea-db.org/help.

#### Rhea reaction pages

**Figure 1.**
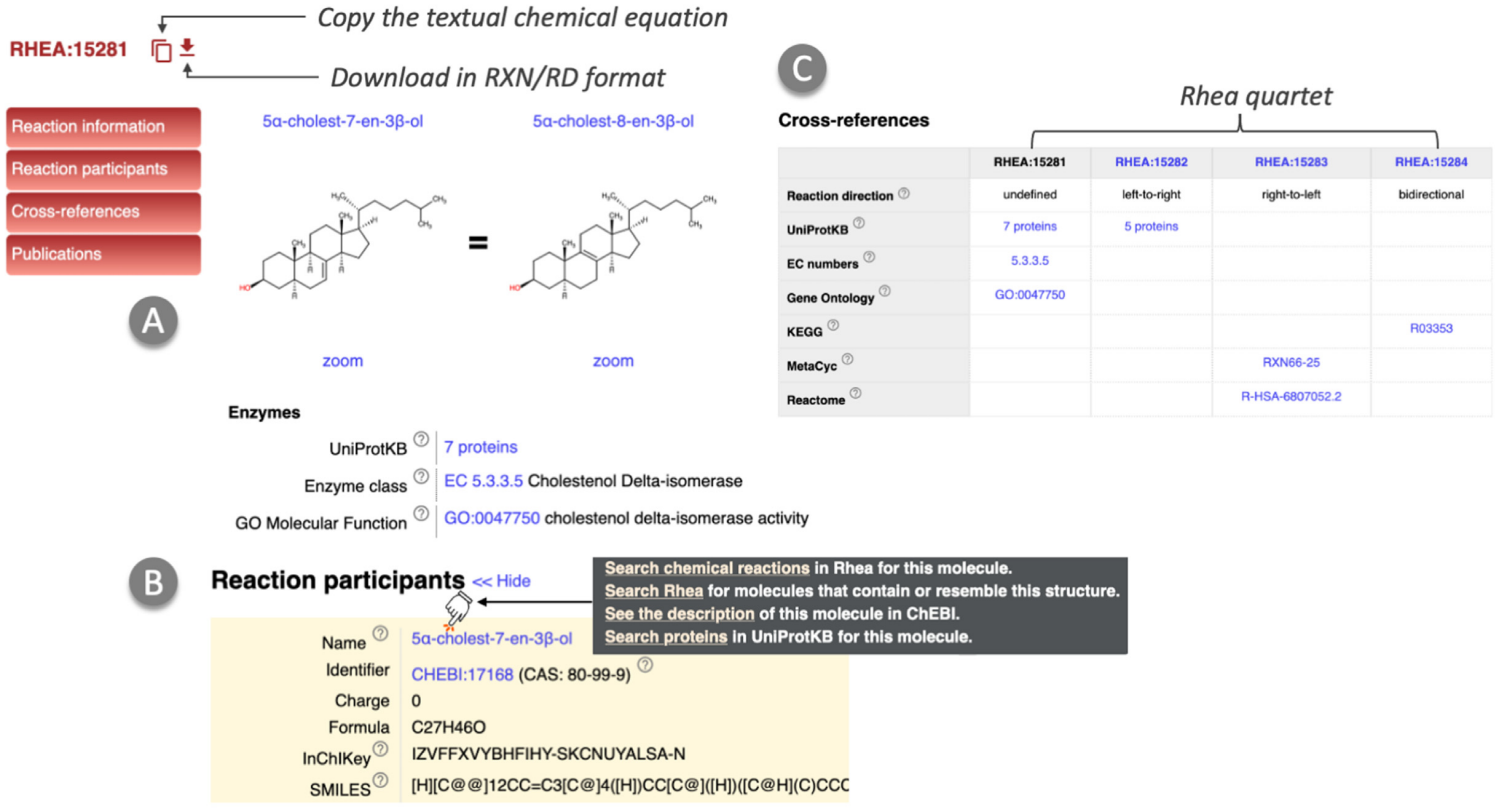
Rhea Reaction page. Each reaction page contains sections providing information on the reaction itself and associated enzymes, as well as reaction participants, cross references, publications, and other information such as comments. Action buttons allow users to copy the reaction equation and download the directed forms of reaction in RXN and RD formats, while a multi-faceted tooltip linked to the name of each participant allows users to launch searches in Rhea, ChEBI and UniProtKB. (**A**) reaction information section, (**B**) detailed information regarding reaction participants, (**C**) cross-references for each member of the Rhea quartet (each member corresponds to different directions but the same transformation).

The Rhea reaction pages consist of several sections (Figure 1). The *Reaction information* section (Figure 1A) provides a graphical representation of the reaction (including chemical structures of reaction participants) served by a web component that developers can embed in their own webpages using our npm package (https://www.npmjs.com/package/@swissprot/rhea-reaction-visualizer). This section also includes information on enzymes in the form of links to the corresponding enzyme classes (EC number(s)), enzyme sequences (UniProtKB protein sequence records), and the corresponding Gene Ontology (GO) term (where a mapping of Rhea to GO is available from the GO Consortium) (29). The *Reaction participants* section (Figure 1B) provides information on each reaction participant, including name, identifier, charge, formula, InChIKey (a simple hash representation of chemical structures that encodes information on connectivity, stereochemistry and charge in three distinct blocks) (https://www.inchi-trust.org) (30), SMILES (Simplified Molecular-Input Line-Entry System) (http://opensmiles.org), a linear notation for chemical structures, and 2D structure coordinates (MDL Molfile). In addition, this section allows users to launch searches in Rhea for reactions involving each participant via a multi-faceted tooltip linked to the name. The *Cross-references* section (Figure 1C) provides links to relevant information from a range of resources including reactions from KEGG (31), Reactome (32), MetaCyc (33), EcoCyc (34), and M-CSA (35), and enzyme information from UniProtKB, the Enzyme Classification, and the GO, and indicates to which member of the quartet of Rhea reaction identifiers each cross-reference applies (Figure 1C). The remaining sections are *Related reactions*, which provides a list of reactions that are either general or specific forms of the current reaction (parent/child reactions), *Publications*, which lists the peer-reviewed literature from which the reaction was curated, and *Comments*, which provides additional information about the reaction. The Rhea reaction page also features action buttons (at the top of each page) that allow users to copy a textual representation of the reaction equation on a clipboard or download the reaction in RXN or RD format.

#### Searching Rhea

**Figure 2.**
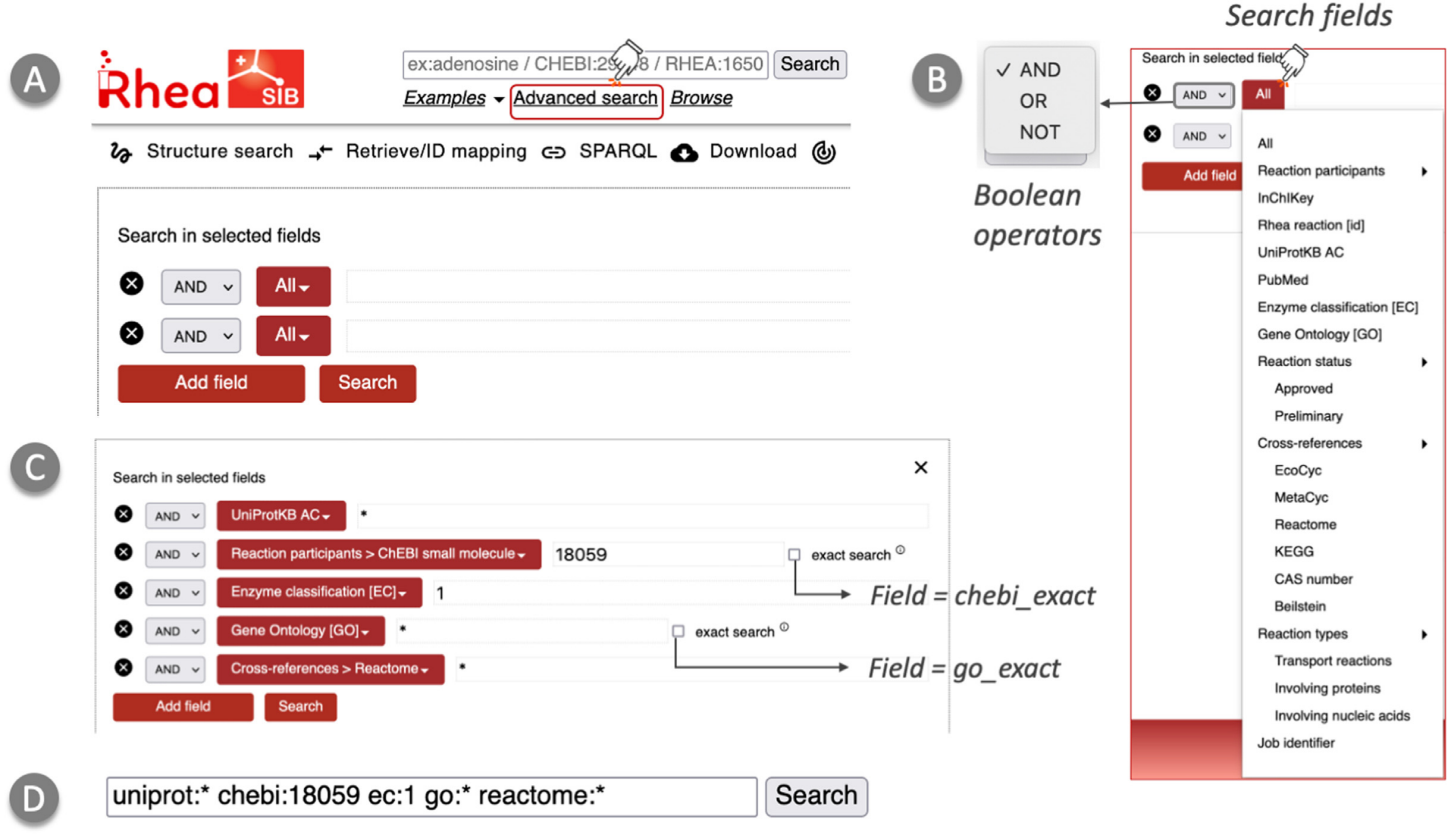
Rhea Advanced Query builder. (**A**) The Rhea query builder can be accessed by clicking on the *Advanced search* link. (**B**) Clicking on the *All* button allows users to access a list of searchable fields and select Boolean operators (see Supplementary Table 1). (**C**) A complex query can be constructed using multiple constraints. An *exact search* option is provided for searches using identifiers from ChEBI and the Gene Ontology. This option allows users to perform searches without using the ‘*is a*’ relationships. (**D**) Final query created using the query builder.

Users can search Rhea by providing search strings that may include chemical names, chemical identifiers (ChEBI, Beilstein & CAS numbers) and reaction identifiers (KEGG, Reactome, MetaCyc, EcoCyc and M-CSA), enzyme classes (EC numbers), UniProtKB accession numbers, GO term identifiers, and PubMed identifiers. Clicking on *Advanced search* opens the query builder (Figure 2A), which allows users to select specific search fields (which is useful for disambiguation) and to combine searches in fields using Boolean operators AND, OR, NOT (Figure 2B). The query builder also provides an autocomplete feature for some fields (ChEBI names and GO Molecular Function terms) that helps users to choose among a number of related possibilities as they type. We provide an exhaustive list of the query fields used in Rhea in Supplementary Table 1.

Certain search fields in Rhea contain terms from ontologies such as ChEBI and the GO. Rhea indexes the ‘*is a*’ relationships from both ChEBI and the GO, such that a user who searches Rhea using an identifier from either ontology will be presented by default with all reactions involving (or mapped to) members of that class, and of any child class. Hence, a search for ChEBI:18059, the class of lipids, will return all reactions involving any type of lipid. Users who wish to override this default behavior, and search using ChEBI and GO identifiers without considering the ‘*is a*’ relationships, can do so by selecting the *Exact* option in the *Advanced search* interface (Figure 2C). Users can also search for all reactions that map to a specific level of the enzyme classification using partial EC numbers, such as ‘ec:2’, ‘ec:2.1’ or ‘ec:2.1.1’.

The following query illustrates a number of the aforementioned features, combining multiple fields using Boolean operators, searching ontologies and hierarchical vocabularies, and using resource names, identifiers, and wildcards. This query retrieves all reactions annotated in UniProtKB that involve lipids, that map to EC class 1 (Oxidoreductases) and that have a mapping to the GO and Reactome (Figure 2C and D):

UniProt:* AND ChEBI:18059 AND EC:1 AND GO:* AND Reactome:*

We close this section with a brief description of how Rhea handles protonation states in ChEBI. The ChEBI ontology treats each protonation state of a given chemical entity as a distinct entity, with its own unique identifier. Rhea selects only one protonation state for each chemical entity by default, that which represents the major protonation state at pH 7.3. The Rhea search engine maps ChEBI identifiers for other protonation states to the major protonation state used in Rhea, using a precomputed mapping of chemical structures available at https://www.rhea-db.org/help/download. This applies to *Exact* searches too, the logic being that if other protonation states were not mapped there would be no results.

#### Chemical structure search in Rhea

Users can search for Rhea reactions using complete or partial InChIKeys through the *Advanced search* or the simple search interfaces (using the prefix ‘inchikey:’). Users can also map lists of (complete or partial) InChIKeys to Rhea reactions using the bulk retrieve/ID mapping functionality described below.

#### Chemical similarity and substructure search in Rhea

The Rhea website supports searches for reactions that involve a compound that is either similar to, or a derivative of, a given compound of interest, via the structure search interface at https://www.rhea-db.org/structuresearch. Rhea uses the IDSM Sachem chemical cartridge for both fingerprint-guided similarity and substructure searches (36). Users can provide query structures in the form of valid SMILES or by importing or drawing structures using the Ketcher molecular editor provided (https://lifescience.opensource.epam.com/ketcher).

#### Search results

**Figure 3.**
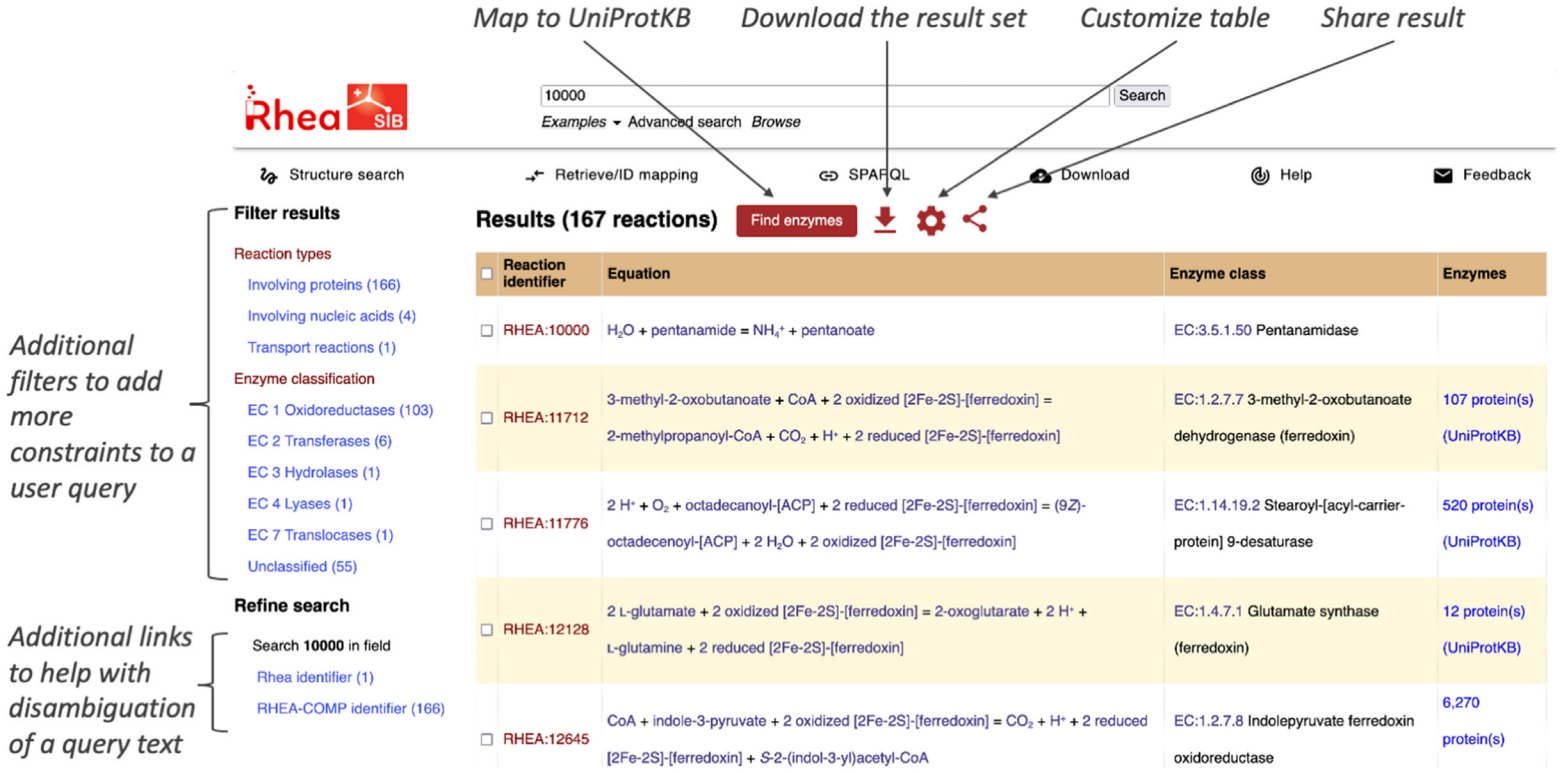
Search Results. Search results are displayed in tabular form, where each row corresponds to a single reaction. Action buttons (top) allow users to map selected reactions to UniProtKB, to download them, to add or remove columns from the result table, or to create a link (URL) for the result that can be bookmarked, shared and reused.

The Rhea website presents search results in tabular form, where each row corresponds to a single reaction and each column corresponds to one aspect of a particular reaction (Figure 3). Action buttons allow users to add or remove columns from the result table, to download all or selected results in a range of formats, to map a selection of the reactions to UniProtKB, and to create a link for the result that can be bookmarked, shared and reused (see section ‘*Programmatic access’* below). Users can apply additional constraints to the result set by clicking on filters in the left side navigation panel, limiting the results to specific types of reaction participants (such as proteins), specific types of reactions (such as transport reactions), or specific enzyme classes. A *Refine search* feature lists relevant fields that users can select when using query strings that match multiple fields. The chemical similarity and chemical substructure searches do not return a list of reactions but rather a list of ChEBI compounds that match the query structure and that are participants in Rhea reactions.

#### Identifier mapping

**Figure 4.**
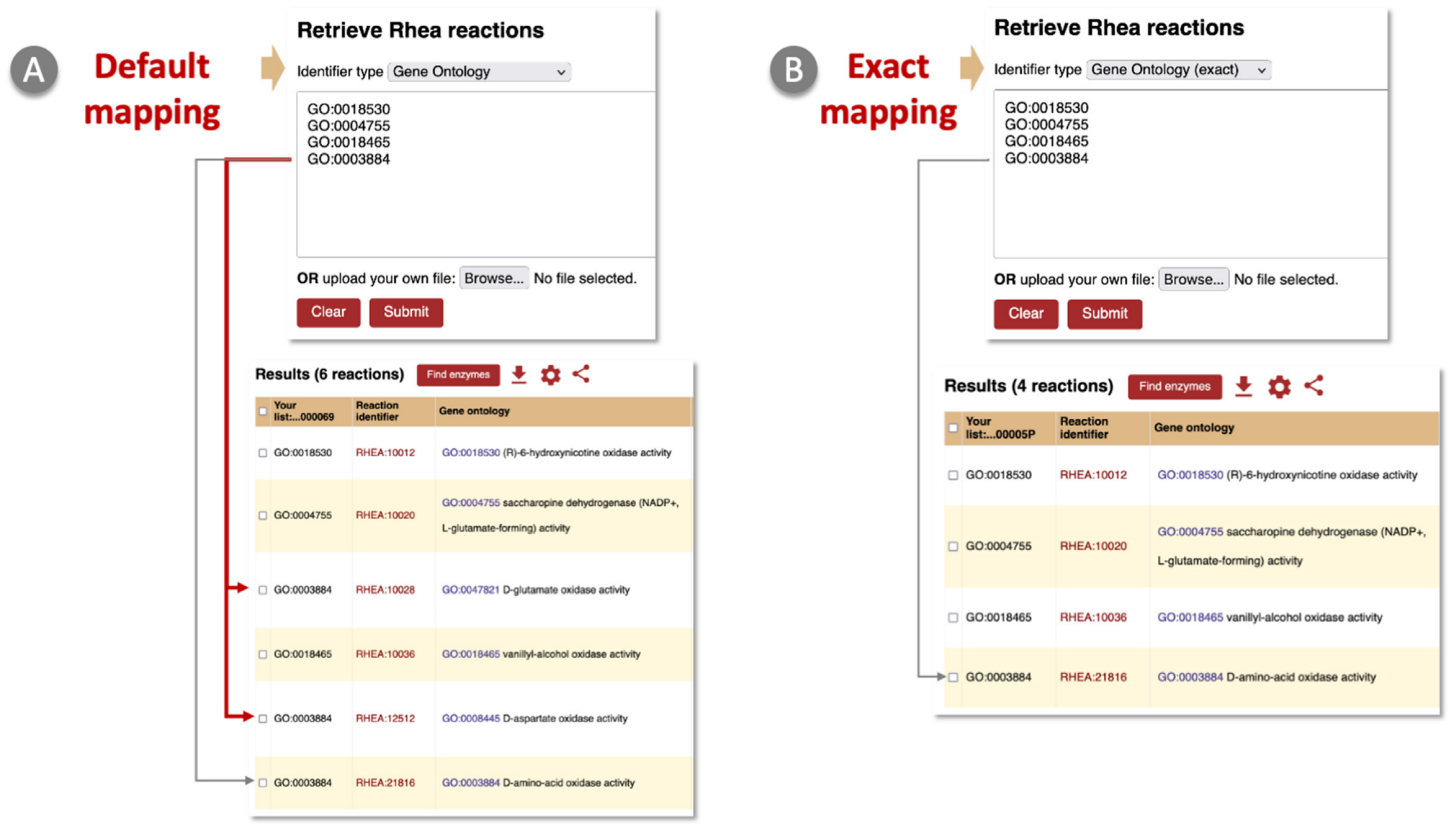
Identifier mapping. (**A**) By default, *Gene Ontology* identifier mapping will retrieve Rhea reactions mapped to a user supplied GO term and to all children of that GO term. In this example, the user supplied GO term, GO:0003884 (D-amino-acid oxidase activity), maps directly to RHEA:21816 (grey arrow) as well as to RHEA:12512 (via GO:0008445, D-aspartate oxidase activity) and RHEA:10028 (via GO:0047821, D-glutamate oxidase activity) (red arrows). (**B**) The *Gene Ontology (exact)* search option limits mapping to Rhea reactions directly mapped to GO:0003884 (D-amino-acid oxidase activity)—RHEA:21816—and excludes reactions mapped to GO terms that are children of GO:0003884.

The Rhea identifier mapping service (https://www.rhea-db.org/mapping) accepts identifiers for reactions from KEGG, Reactome, MetaCyc, EcoCyc and M-CSA, identifiers for chemical entities (currently ChEBI identifiers and the InChIKey), and identifiers for enzyme classes and functions (currently EC numbers and identifiers from the Molecular Function branch of the GO). Users can copy-paste a list of identifiers, or upload a file containing a list of identifiers, and specify the input identifier type, in order to obtain a mapping of their identifiers to Rhea reactions. As with the *Advanced search* feature, hierarchical mapping is performed by default for GO and ChEBI, in which a user specified term is mapped to all Rhea reactions mapped to child terms in the relevant ontology (Figure 4A), but the drop-down menu also provides an *Exact* mapping option (Figure 4B). The Rhea identifier mapping service provides results in the standard result table, with an additional column for each row that lists the user-provided identifier(s) that were mapped to that Rhea reaction (one Rhea reaction per row). The identifier mapping tool also accepts Rhea reaction identifiers, which allows users to retrieve a list of Rhea reactions in tabular format.

#### Programmatic access

Rhea provides programmatic access to all data, queries and tools available through the Rhea website via RESTful URLs that users can bookmark, link, and use in their own programs. The easiest way to create a URL is using our interactive *Advanced query* builder, and then use the *Share* icon to obtain the URL to which the requisite *format* parameter can be added. Individual reaction data is available in RXN and RD formats, with search results in customizable tab-separated formats. Supplementary Table 2 provides the possible query parameters for a REST request and Supplementary Table 3 provides the list of possible columns to customize the result set. The examples shown below - for Unix and Python 3 - use the REST API to retrieve all Rhea reactions mapped to UniProtKB enzyme sequences and to export them as a tab-delimited file containing Rhea reaction identifiers, reaction equations, and UniProt accession numbers.

**Table:** 

Unix:
curl ‘https://www.rhea-db.org/rhea/?query=uniprot:*&columns=rhea-id,equation,uniprot&format=tsv&limit=10’ -o test.tsv
Python 3:
import requests
url = ‘https://www.rhea-db.org/rhea?’
parameter = {
‘query’:‘uniprot:*’,
‘columns’:‘rhea-id,equation,uniprot’,
‘format’:‘tsv’,
‘limit’:10,
}
response = requests.get(url,params = parameter)

### Rhea SPARQL endpoint

The Rhea SPARQL endpoint (https://sparql.rhea-db.org/sparql) supports complex and federated queries over Rhea RDF and RDF of other resources providing SPARQL endpoints through federated queries, including chemical similarity and substructure searches using the IDSM/Sachem SPARQL endpoint (37). We invite interested readers to consult the SPARQL endpoint and sample queries and documentation provided there.

### Selection of Rhea as an ELIXIR Core Data Resource

ELIXIR (https://elixir-europe.org) (38) works to link national centres and core bioinformatics resources in Europe into a single coordinated infrastructure for life science data. ELIXIR has created a formal process to identify the most critical life science data resources in Europe, which are termed ELIXIR Core Data Resources (CDRs) (39,40). ELIXIR selected Rhea as an ELIXIR Core Data Resource in 2021. There are currently 22 ELIXIR Core Data Resources (CDRs), covering genes and genomes, proteins, small molecules, molecular structures, interactions, and literature. Rhea is the first ELIXIR CDR that focuses on reactions and bridges UniProt, the ELIXIR CDR for proteins, and ChEBI, the ELIXIR CDR for small molecules.

## DISCUSSION

Rhea is a reference resource of computationally tractable enzyme and transport reaction data and the standard for enzyme and transporter annotation in UniProtKB. The Rhea website, API and SPARQL endpoint provide a powerful toolbox to mine the Rhea reaction dataset for a broad range of applications, while the UniProt website, API and SPARQL endpoint allow users to exploit the rapidly growing set of enzyme and transporter annotations created using Rhea. Perhaps one of the most exciting applications of Rhea (and UniProt) data to emerge recently is the use of machine intelligence to study and design enzymes and biosynthetic and bioremediation pathways—combining state of the art deep learning models of language to describe both protein sequences (41–46) and small molecule chemistry (47–50). We will continue to develop the Rhea dataset, website and modes of programmatic access to better support these and other applications, and will continue to work with other key knowledge resources such as UniProt, the GO and Reactome to improve the consistency and interoperability of enzyme, transporter, and reaction data in all of our resources (51,52).

## DATA AVAILABILITY

The Rhea website is available at https://www.rhea-db.org/ and the Rhea SPARQL endpoint at https://sparql.rhea-db.org. All data in Rhea is freely available under a Creative Commons Attribution License (CC BY 4.0) and users can download it from our FTP site https://ftp.expasy.org/databases/rhea/ in the following formats: RDF, BioPAX, RXN/RD, and TSV. More information regarding different downloadable files provided by Rhea is available at https://www.rhea-db.org/download. We now synchronize Rhea and UniProtKB releases (as of February 2020), which are published approximately every eight weeks. We provide a snapshot of the ChEBI data corresponding to the Rhea and UniProtKB release on the Rhea ftp site, as well as an export of the 2D structures in MOL and SDF formats. Video tutorials on how to use Rhea website are freely available on the SIB (Swiss Institute of Bioinformatics) YouTube channel https://www.youtube.com/channel/UCPo4ED_WAKjwQ878cca6_oQ - we list available videos in Supplementary Table 4. Users that would like to have reactions added to Rhea are very welcome to use the Feedback form (https://www.rhea-db.org/feedback) - input from our users is highly appreciated.

## Supplementary Material

gkab1016_Supplemental_File
