# Supplementary material for: Rhea, the reaction knowledgebase in 2022
Source: Nucleic Acids Res. 2021 Nov 10;50(D1):D693–700. doi: 10.1093/nar/gkab1016 (PMC8728268; doi:10.1093/nar/gkab1016)
Supplement: gkab1016_Supplemental_File [file gkab1016_supplemental_file.pdf]

Field name	Example query	Query builder field > subfield label	Description
chebi, chebi_exact	chebi:59871, chebi_exact:59871	Reaction participants > ChEBI small molecule	Search small molecule by name, synonym, chemical class or identifier
rhea-comp	rhea-comp:10594	Reaction participants > Rhea macromolecule	Search macromolecules (proteins, nucleic acids or Rhea polymer) by name or identifier
inchikey	inchikey:VFRROHXSMXFLSN	InChIKey	Search by full or partial InChIKey
rhea	rhea:42796	Rhea reaction [id]	Search by Rhea identifier
uniprot	uniprot:P32483	UniProtKB AC	Search by UniProtKB accession
pubmed	pubmed:29867142	PubMed	Search by PubMed identifier
ec	ec:2.1.1.160	Enzyme classification [EC]	Search by complete or partial EC number
go, go_exact	go:0003884, go_exact:0003884	Gene Ontology [GO]	Search by Gene Ontology (GO) 'Molecular Function' name or identifier
status	status:approved	Reaction status > Approved	Search for reactions that are approved
status	status:preliminary	Reaction status > Preliminary	Search for reactions with limited experimental evidence (may include unbalanced reactions for mass and charge)
ecocyc	ecocyc:PABASYN-RXN	Cross-references > EcoCyc	Search by EcoCyc reaction identifier
metacyc	metacyc:PABASYN-RXN	Cross-references > MetaCyc	Search by MetaCyc reaction identifier
reactome	reactome:R-HSA-193706.1	Cross-references > Reactome	Search by Reactome reaction identifier (limited to Hsa)
kegg	kegg:R01716	Cross-references > KEGG	Search by KEGG reaction identifier
macie	macie:M0283	Cross-references > M-CSA/MACiE	Search by M-CSA/MACiE reaction identifier
cas	cas:57-88-5	Cross-references > CAS number	Search by CAS Registry Number
beilstein	beilstein:2060565	Cross-references > Beilstein	Search by Beilstein Registry Number
transport	transport:yes	Reaction types > Transport reactions	Search for transport reactions
hasprotein	hasprotein:yes	Reaction types > Involving proteins	Search for reactions with participants that are proteins
hasnucleotide	hasnucleotide:yes	Reaction types > Involving nucleic acids	Search for reactions with participants that are nucleic acids

Supplementary Table 1: List of searchable fields in Rhea

Parameter	Values	Description
query	*string*	Query string. An empty query string will retrieve all entries in a data set
columns	comma-separated list of column names	Columns to include in the result (see Supplementary Table 3) (optional)
format	tsv	Result format: tsv returns data for the selected columns as tab-separated values
limit	*integer*	Maximum number of results to retrieve (optional)

Supplementary Table 2: Parameters that users can add to the URL for accessing Rhea search results.

Column label	Column ID	Value
Reaction identifier	rhea-id	Reaction identifier (with prefix RHEA)
Equation	equation	Textual description of the reaction equation
ChEBI name	chebi	Semicolon-separated list of ChEBI names used as reaction participants
ChEBI identifier	chebi-id	Semicolon-separated list of ChEBI identifiers used as reaction participants
EC number	ec	Semicolon-separated list of EC numbers (with prefix EC)
Enzymes	uniprot	Number of proteins (UniProtKB entries) annotated with the Rhea reaction
Gene Ontology	go	GO identifier (with prefix GO) and label
PubMed	pubmed	semicolon-separated list of PubMed identifiers (without prefix)
Cross-reference (EcoCyc)	reaction-xref(EcoCyc)	EcoCyc reaction identifier
Cross-reference (MetaCyc)	reaction-xref(MetaCyc)	MetaCyc reaction identifier
Cross-reference (KEGG)	reaction-xref(KEGG)	KEGG reaction identifier
Cross-reference (Reactome)	reaction-xref(Reactome)	Reactome reaction identifier
Cross-reference (M-CSA/MACiE)	reaction-xref(M-CSA)	M-CSA reaction identifier

Supplementary Table 3: Column names that users can add to the query URL for programmatic access.

Topic	YouTube URL
The Rhea knowledgebase: key features & web interface – Guided tour #1	https://www.youtube.com/watch?v=XDcN8L5KqS8
The Rhea knowledgebase: web interface & reaction pages – Guided tour #2	https://www.youtube.com/watch?v=yWi3e7xbp_A
How to search for Rhea reactions involving specific chemical compounds	https://www.youtube.com/watch?v=0tow_vBNir8
How to search for the enzymes corresponding to a Rhea reaction in the UniProt knowledgebase	https://www.youtube.com/watch?v=n8X2b4uwN0Y
How to search for Rhea reactions by enzyme class (EC number)	https://www.youtube.com/watch?v=yGbF8N2hqkk
How to search for Rhea reactions with the Gene Ontology (GO)	https://www.youtube.com/watch?v=uNYlMknEYok

Supplementary Table 4: List of Rhea videos (guided tours and tutorials) available on the SIB YouTube channel.
